# Supplementary material for: PRE-POET: a qualitative interview-based study to explore patient-relevant factors in the context of prostate biopsy
Source: BMC Urol. 2025 Nov 28;25:299. doi: 10.1186/s12894-025-02000-5 (PMC12667091; doi:10.1186/s12894-025-02000-5)
Supplement: Supplementary file 2 — Supplementary Material 2. [file 12894_2025_2000_MOESM2_ESM.docx]

**
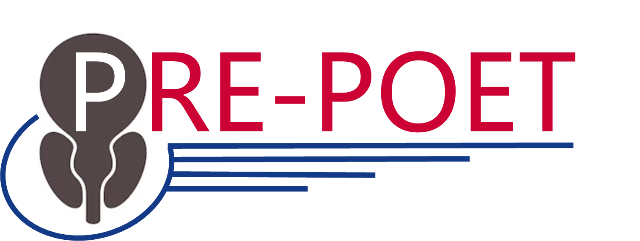
**

**Interview guideline patient interviews**

| 1. **Welcome & Introduction** |
| --- |
| Thank you for your willingness to participate in our study. My name is XX, I am a research associate at the Institute of Medical Biometry and Statistics, Section of Health Services Research and Rehabilitation Research at the University Medical Center Freiburg. You have received written patient information in advance and have consented to us recording this interview for research purposes. All names and information that can be traced back to you as a person will be made unrecognizable and pseudonymized.  According to my information, you recently visited the urology department at the UKF for a consultation about an upcoming prostate biopsy, which will be performed on you shortly. I would like to talk to you today about your thoughts on this subject. In this context, it is important that you say everything you think. There is no right or wrong and nothing you tell me will influence your treatment. Your treating doctors will not know anything about the content of our conversation.  Please note that I am not a doctor myself. If you have any medical questions during or after our consultation, you can contact the doctor treating you at any time. If our conversation gives you any negative thoughts, please let me know at any time. The University Hospital also offers a psycho-oncological counseling service in such cases. You can find the telephone number in the patient information leaflet that was given to you. Otherwise I will be happy to give it to you again. |

| 1. **Transition** |
| --- |
| Let's start by talking about how today's telephone appointment came about.  What motivated you to talk to me today?   - Why did you decide to take part in this interview? - Can you pin this down to any particular key points?   What do you think could be the reasons for patients in your situation not to take part in this survey?   - Why are these reasons not relevant to you? |

| 1. **Fears in the context of prostate biopsy and cancer** |
| --- |
| At the beginning of our telephone conversation, we had already talked about the fact that you are due to have a prostate biopsy soon. This procedure is done to check whether a tumor is growing in the prostate or not.  What feelings and thoughts do you have when you hear the word "prostate tumor" or "prostate cancer"?   - What fears or worries do you have in this context? - What would help to reduce these fears?   You were told how the biopsy would be performed.   - How did you feel about the conversation? - To what extent do you feel well informed about the prostate biopsy as a result of the discussion at the clinic?   What fears or worries do you have when you think specifically about this procedure?   - What should we or the clinic do to alleviate your concerns about this procedure? - Why did you decide to have this procedure? - In your opinion, are there any reasons why you should not have this biopsy?   A prostate biopsy can be performed under general or local anesthesia. General anesthesia carries certain risks and also leads to longer waiting times in the clinic. Nevertheless, there are patients who prefer this type of procedure.  How is it with you?   - Why did you opt for a local anesthetic biopsy? - What factors were important to you? |

| 1. **Diagnostic precision and side effects** |
| --- |
| You have already been informed by the doctor about the procedure, i.e. the prostate biopsy, which you are about to undergo.  What side effects are you aware of in this context?   - What side effects do you think you will experience? - Which ones would you be more or less able to accept? - Are there any side effects that you would not accept at all or that you are particularly concerned about?   According to our practicing doctors, prostate biopsies can have side effects that are only temporary. These can include: inflammation of the prostate, bleeding, pain when sitting, nocturnal urination, a strong urge to urinate, worsening of urinary incontinence or erectile dysfunction.   - (If nothing has been mentioned before): Which of these side effects would you be more able to accept and which would you be less able to accept?   A prostate biopsy, i.e. the removal of tissue to test for the presence of cancer, is a diagnostic procedure. Such procedures always have a certain degree of inaccuracy. The informative value of the examination can be increased by taking more tissue samples. However, the risk of side effects also increases with the number of samples taken. It is therefore always a question of weighing up the risk and the certainty of the diagnosis.  You are now in a situation where there are two possibilities: either you have a tumor or you don't.  How do you feel about this, would you rather have fewer side effects and be less certain that the tumor (if present) will be found, or would you rather accept more side effects or stress and be more certain of the diagnosis?   - Can you explain your thoughts on this?   Suppose there is a new procedure which, according to statistical tests, has the same probability of finding cancer. The only difference is that the MRI, i.e. the image of the prostate via MRI, is done a little differently and therefore a few fewer samples could be taken.   - What do you think, could you imagine preferring this new procedure to the old one? - In your opinion, what are the reasons for/against it? |

| 1. **Willingness to participate in POET** |
| --- |
| We are currently planning a new clinical study, which will also be carried out at the UKF in the future. The aim of our study is to improve the diagnostic accuracy of a prostate biopsy. This means that the biopsy, i.e. the removal of tissue samples from the prostate, can be used to more accurately assess whether cancer is present or not. Specifically, the aim is to use a new procedure to select the areas of the prostate to be examined in a more targeted manner. In order to test the effectiveness of this procedure, a clinical study must be carried out to compare it with the standard procedure that is normally used. In the best case scenario, this new method is suitable for the same diagnostic accuracy, so that fewer samples need to be taken and therefore fewer side effects occur. However, it is also possible that this method is not as accurate as the method currently in use, in which case it will not be introduced into practice.  Would you be willing to participate in such a study?   - What are your reasons / thoughts on this?   What do you think this study would need to look like for you to participate?  How do you think we could design this study so that it meets the wishes and needs of patients in your situation? |

| 1. **Communication preferences** |
| --- |
|  |

| What questions or information are particularly important to you when it comes to prostate biopsies and cancer?  Were you able to clarify all your questions with your doctor at the university hospital?   - What was particularly important to you? - Have any new questions arisen since your consultation at the university clinic? - What are they? - Is there anything about the way you were informed by the doctor that you think could be improved?   How do you deal with it now? |
| --- |
| 1. **Further outcomes** |
| You have already told me many things. We would like to incorporate the opinions of patients in future studies and investigations into prostate biopsies and prostate cancer.  Are there any other open points that we have not yet addressed but that you would like to get rid of?   - Follow-up: Is there anything that is important to you, from the information session to the biopsy to the further treatment of a possible cancer? |

Thank you for sharing your thoughts with me.

If you have any uncertainties, worries or fears after our conversation, please do not hesitate to contact us. You can either contact the doctor treating you, the psycho-oncological counseling center or, of course, me.

You will find the contact details on the patient information leaflet that was given to you.

I wish you all the best for your biopsy and would like to thank you once again for agreeing to talk to me in this situation.
