# Supplementary material for: PRE-POET: a qualitative interview-based study to explore patient-relevant factors in the context of prostate biopsy
Source: BMC Urol. 2025 Nov 28;25:299. doi: 10.1186/s12894-025-02000-5 (PMC12667091; doi:10.1186/s12894-025-02000-5)
Supplement: Supplementary file 3 — Supplementary Material 3. [file 12894_2025_2000_MOESM3_ESM.docx]

PRE-POET: A qualitative interview-based study to explore patient-relevant factors in the context of prostate biopsy

PRE-POET: A qualitative study on prostate biopsy

Jan G. Halbich^1*^ [0009-0002-3013-3181](https://orcid.org/0009-0002-3013-3181), jan.gero.halbich@uniklinik-freiburg.de

August Sigle^1^, [0000-0001-6857-8149](https://orcid.org/0000-0001-6857-8149), august.sigle@uniklinik-freiburg.de

George Jogho^2^, [0009-0000-3268-6324](https://orcid.org/0009-0000-3268-6324), george.jogho@uniklinik-freiburg.de

Erik Farin-Glattacker^2^, [0000-0001-6867-0316](https://orcid.org/0000-0001-6867-0316), erik.farin@uniklinik-freiburg.de

Gabriele Dreier^3^, [0009-0008-7908-8258](https://orcid.org/0009-0008-7908-8258), gabriele.dreier@uniklinik-freiburg.de

Urs A. Fichtner^2^, [0000-0002-4190-8969](https://orcid.org/0000-0002-4190-8969), urs.fichtner@uniklinik-freiburg.de

^1^ Department of Urology, Faculty of Medicine, University of Freiburg - Medical Centre, Freiburg, Germany

²Section of Health Care Research and Rehabilitation Research, Institute of Medical Biometry and Statistics, Faculty of Medicine, Medical Center, University of Freiburg, Freiburg, Germany

^3^ Clinical Trials Center, Medical Center - University of Freiburg, Freiburg, Germany

*Corresponding Author

**Statements and declarations**

**Data availability**

The data that support the findings of this study are available on request from the corresponding author. The data are not publicly available due to privacy or ethical restrictions.

**Funding**

The funding for this study was provided by the program for clinical studies by the Medical Center – University of Freiburg, Faculty of Medicine, University of Freiburg.

**Competing interests**

The authors declare that they have no conflicting interests.

**Ethics approval and patient consent to participate**

Ethics approval for this study was granted by the ethics committee of the University of Freiburg (Reference number: 24-1101-S1, 16.04.2024). Written informed consent was obtained from each patient in advance.

**Consent for publication**

All the authors have approved the final version of the manuscript.

**Authors' contributions**

JH: conceptualization, methodology, investigation, resources, project administration, writing - original draft. AS: conceptualization, resources, supervision, funding acquisition. GJ: software, validation, data curation, writing – review & editing. EF: Resources, supervision, writing – review & editing. GD: conceptualization, methodology, writing – review & editing. UF: conceptualization, methodology, software, validation, formal analysis, investigation, data curation, visualization, project administration, writing – original draft

**Acknowledgments**

We want to thank all the patients who contributed to this work.

**Clinical trial registration**

This study was registered at the Freiburg Clinical Trial Register (FRKS005027).

**Use of artificial intelligence-generated content (AIGC) tools**

The authors disclose the use of generative AI and AI-assisted technologies in the writing process for translation and paraphrasing.

**List of abbreviations**

AS – Active surveillance

iPC – Indolent prostate cancer

mpMRI – Multiparametric Magnetic Resonance Imaging

MRI – magnetic resonance imaging

PCa – Prostate cancer

PLT – Perilesional template

PPI – Patient and public involvement

PROMs – Patient-reported outcome measures

PSA – Prostate-specific antigen

SB – Systemic biopsy

sPC – significant prostate cancer

TB – targeted biopsy

**Abstract**

**Introduction:**

Prostate biopsy is a key diagnostic tool for prostate cancer and is often associated with physical, emotional, and social challenges. To enhance future multicenter trial designs for diagnostic precision, understanding patient-relevant outcomes is essential. Current evidence on patient-reported outcomes (PROMs) and patient-reported experience measures (PREMs) in the context of prostate biopsies remains limited, particularly regarding perioperative biopsychosocial impacts. This study aimed to explore patient-relevant factors, including concerns, side effect tolerance, and preferences, to inform future trials through patient and public involvement (PPI).

**Methodology:**

A prospective monocentric qualitative study was conducted with 12 male patients (mean age: 69.8 years) who underwent prostate biopsy. Preprocedure semistructured telephone-based interviews were conducted from May to August 2024 in Freiburg, Germany, transcribed verbatim, and analyzed via deductive-inductive content analysis. Intercoder reliability reached a kappa of 0.73. A short questionnaire was used to collect background data. The interviews addressed themes such as study motivation, fears, diagnostic precision, side effects, communication preferences, and other outcomes relevant to patients.

**Results:**
The participants expressed fears about physical side effects (e.g., pain, incontinence, and erectile dysfunction) and potential cancer diagnoses. While most patients tolerate temporary side effects for the sake of diagnostic accuracy, potential long-term effects raise concerns. Trust in the medical team’s expertise, the reputation of the medical center, and transparent communication were identified as crucial for patient satisfaction. Many participants showed altruistic motivation to contribute to research but emphasized the importance of shorter waiting times and clear communication regarding biopsy risks and benefits. The biopsychosocial model was evident, as patients reported interconnected physical, psychological, and social burdens. Preferences for open, empathetic communication and reduced procedural invasiveness were recurrent themes.

**Conclusions:**
This study highlights the multidimensional nature of patient experiences with prostate biopsy, emphasizing the importance of patient-centered research. Transparent communication, trust, and minimizing invasiveness while ensuring diagnostic accuracy are essential for improving patient satisfaction and reducing anxiety. The findings provide valuable insights for designing future studies and underline the importance of incorporating patient-relevant outcomes in clinical research and decision-making.

**Key Points:**

Perioperative prostate biopsies impose physical, psychological, and social stress on patients, consistent with the biopsychosocial model. Patient satisfaction and willingness to participate in studies are strongly influenced by trust in medical expertise and transparent, empathetic communication. While patients are primarily motivated by altruism, they also expect high diagnostic accuracy and are willing to tolerate only transient side effects.

**Keywords:**

Adverse effects, Image-Guided Biopsy, Diagnostic Techniques and Procedures, Patient Involvement, Patient Satisfaction, Patient Reported Outcome Measure, Prostatic Neoplasm

This study was registered at the University Medical Center of Freiburg Clinical Trial Register (FRKS005027).

**1. Introduction**

- 1. **Prostate cancer diagnostics**

In 2022, prostate cancer ranked as the second most prevalent cancer among men globally, with 1.5 million new diagnoses, and was the fifth leading cause of cancer-related mortality in men [1]. In Germany, it was the most frequently diagnosed cancer, accounting for 65,296 new cases or 20.2% of all cancer diagnoses [2].

A structured screening program does not currently exist in Germany because of the heterogeneous data regarding its benefits. However, the German prostate cancer guidelines recommend that men over the age of 45 years and with a life expectancy of more than 10 years should be informed about the advantages and disadvantages of a screening examination. If patients choose to pursue this procedure, measuring prostate-specific antigen (PSA) should be recommended. PSA is a tumor marker that may be elevated not only in prostate cancer but also in cases of benign prostatic hyperplasia or inflammation [3].

In patients with suspected significant prostate cancer, multiparametric magnetic resonance imaging (mpMRI) of the prostate is typically performed before a biopsy to improve diagnostic accuracy. On this basis, a biopsy decision can be made, and a targeted biopsy (TB) of suspicious lesions can be performed [4, 5]. Prostate cancer is clinically and histologically classified into indolent prostate cancer (iPC), which typically remains asymptomatic and does not contribute to mortality, and significant prostate cancer (sPC), which is defined by a Gleason score of 7a or higher, with potential harm to and effects on the patient’s life expectancy.

However, even with MRI-targeted biopsy, 25% of significant prostate cancer cases are missed [5–8]. The limited sensitivity of this diagnostic procedure may be attributed to several factors. First, prostate cancer lesions may be undetectable on MRI (technical inaccuracies) or may be missed by radiologists (observer-related diagnostic errors). Second, targeting errors (operator-related inaccuracies, where the biopsy operator fails to sample a lesion that was correctly identified by radiologists) can result in MRI-visible lesions being overlooked. In this context, current guidelines advocate for an additional systematic sector-based biopsy [3, 4]. Therefore, up to 32 additional systematic biopsies are performed alongside the targeted biopsy [9]. On the one hand, these supplementary biopsies have the desired effect in that significant prostate cancer is less frequently missed, thus ensuring greater diagnostic accuracy. On the other hand, however, systematic biopsy also leads to an overdiagnosis of indolent prostate cancer. Tosoian et al. reported that in low-risk PCa patients (iPCs) under active surveillance (AS), the incidence of PCa-specific mortality or metastasis was 0.1% at 10 and 15 years, whereas the incidence rates of biopsy grade reclassification at 5, 10, and 15 years were 21%, 30%, and 32%, respectively [10].

Furthermore, systematic biopsy is associated with increased resource expenditure and a potentially increased complication rate due to the greater number of biopsy cylinders taken.

The challenge in systematic prostate biopsy (SB) is to identify significant prostate cancer (sPC) that may have been missed in MRI-targeted biopsy while simultaneously minimizing the overdiagnosis of iPC. An intuitive approach to solve this problem is the different spatial distributions of sPCs compared with iPCs within the prostate. Previous studies have shown that sPCs are most frequently localized in close proximity to MRI-apparent lesions [11–13]. In a recently published paper, Brisbane et al. analyzed a retrospective cohort of 2,048 men and demonstrated that 90% of sPC-bearing biopsy cylinders were located within a 10 mm radius of the nearest MRI lesion. Furthermore, the authors reported that a more concentrated biopsy could avoid the need for a diagnosis of iPC in 17% of cases [14].

On the basis of these results, systematic biopsy should be confined to a 10 mm margin (penumbra) around the target lesion via a perilesional template (PLT) rather than covering the entire prostate by sector. This approach could increase the detection rate of sPCs while reducing the risk of overdiagnosing iPCs.

In addition, the PLT can reduce the total number of cores to be removed, thereby reducing the number of adverse events with potential impacts on micturition, continence and quality of life.

- 1. **Patient and public involvement**

Patient and public involvement (PPI) aims at actively engaging patients in the design of clinical studies to increase acceptance and participation in clinical studies by fostering an active collaborative partnership between researchers and patients [15–17]. Participant-centered study designs offer the benefits of reduced dropout rates in longitudinal studies, as well as improved outcome quality and patient safety [15, 18]. PPI strategies encompass the integration of the patient perspective in an early research phase, such as the development of research questions, the definition of outcome measures and the dissemination of research findings. In study design, discrepancies often arise between patients' and clinicians' views on important outcomes (18). Therefore, including the patient voice in the definition and development of patient-reported outcome measures (PROMs) and patient-reported experience measures (PREMs) helps to ensure that study designs are better tailored to patients, leading to greater relevance with better appropriateness, more meaningful research findings and identification of aspects that researchers may not have considered. [19–21]. Incorporating individual patients' thoughts, concerns, and experiences prior to a necessary prostate biopsy is important to support the design of future research that aims to improve clinical practice and may result in increased patient satisfaction.

- 1. **Patient-reported experiences and outcomes in prostate biopsy**

Prostate biopsy is considered a necessary intervention for obtaining histological specimens and diagnosing PCa (prostate cancer) [3, 4]; however, it is associated with various types of perioperative biopsychosocial impairments. From a physical perspective, although typically temporary, complications such as pain, urinary infection, bleeding, sepsis, urinary retention and erectile dysfunction may occur [22–25]. Miah et al. reported complication rates associated with template-based prostate mapping, including urinary retention (22.55%), hematuria (94.8%), and urinary infection (9.2%), as well as a decline in sexual function across all five functional domains (Erectile Function, Orgasmic Function, Sexual Desire, Intercourse Satisfaction, and Overall Satisfaction) [26]. Psychosocially, prostate biopsy carries a psychological burden due to the overdiagnosis of indolent prostate cancer (iPC) [27]. However, even the biopsy itself is associated with significant morbidity, including pain, anxiety and discomfort, and the feeling of shame [28]. A systematic review on multiparametric MRI and MRI-guided prostate biopsy identified nineteen patient-reported outcomes in ten included studies [23]. Among these, only one study assessed anxiety as a PROM/PREM and measured preprocedural anxiety in 39% of participants [29]. Health-related quality of life was evaluated in two studies [26, 30], both of which reported no significant change following biopsy. The authors of the review emphasize the need for more evidence regarding the emotional, cognitive, behavioral, and social impacts of biopsies [23]. A discussion paper from 2005 reported considerable psychological stress in men undergoing prostate biopsy due to the fear of potential diagnosis of cancer [31]. The authors suggest that pain in this population is often associated with fear, particularly when the procedure has to be repeated. A study comparing MRI-targeted prostate biopsy in addition to transrectal ultrasound-guided systematic biopsy with systematic biopsy alone reported greater discomfort and anxiety in the targeted group than in the control group, with differences in tolerability and pain. The authors hypothesized that patients may experience increased discomfort and anxiety due to the awareness of areas identified in imaging as suspicious for [32]. Although many instruments and literature exist on PROMs in the context of prostate cancer, cancer treatment [33, 34] and postbiopsy follow-up [32, 35], there is limited evidence regarding patient-reported experience measures (PREMs) specifically related to the biopsy procedure itself. A qualitative interview study conducted in China in 2022 identified four key themes regarding patients' experiences prior to prostate biopsy: (1) fear (concerning pain, potential adverse effects, and the possibility of a cancer diagnosis), (2) the impact of lower urinary tract symptoms, (3) internal conflicts (related to hesitation, regret, and embarrassment), and (4) anticipated lifestyle changes[36].

- 1. **PRE-POET study**

The PRE-POET study is a qualitative, interview-based investigation aimed at exploring patient-relevant factors in the context of prostate biopsy.

In contrast, the POET (Prospective evaluation of a new perilesional template for systematic prostate biopsy) pilot study refers to a previously conducted trial evaluating the diagnostic accuracy of the PLT-based approach (TB + PLT) in comparison with the current gold standard (TB + SB) within an intra-individual design. The forthcoming multicenter study, POET II, will extend this work by comparing the PLT method with the gold standard under randomized conditions in a multicenter design.

Within this project (PRE-POET), we aimed to identify aspects that are relevant for patients undergoing a prostate biopsy to determine outcome measures for the planned multicenter study (POET II). Therefore, we consider the PRE_POET study as a patient and public involvement (PPI) initiative, as the qualitative findings derived from patient interviews serve as a foundation for the subsequent study design (POET II) . The guiding research questions were developed and discussed within the whole study team and are as follows:

1. Which factors influence the willingness to participate in a (clinical) study on prostate biopsy?
2. How do patients experience their involvement in diagnostic decisions in the context of prostate biopsies?
3. What fears do patients experience in the context of prostate biopsies?
4. What side effects are tolerable for patients in the context of prostate biopsies?
5. What communication and information preferences do patients have in the context of prostate biopsies?
6. Are there other study outcomes that are relevant for patients who have not yet received attention?
7. **Methods**

**2.1. Study design**

To answer these research questions, we conducted a prospective monocentric qualitative study supplemented by a short standardized questionnaire to assess background variables with a convenient sample of patients who have a future biopsy performed at the Department of Urology- Medical Centre, Freiburg, Germany. The latter covered questions on gender, prior cancer diagnoses, education, cohabitation, migration background, health status (Background Questionnaire of the International Social Survey Programme [37], quality of life (WHOQOL-BREF) [38] and health literacy (HLS-Q16) [39]. The inclusion criteria were men aged 18 years or older with indication for prostate biopsy and the presence of an MRI-suspected lesion (PI-RADS >3), written informed consent, and sufficient command of German language. The exclusion criteria were the absence of an MRI-suspected lesion (PI-RADS >3), previously diagnosed PCa, and anatomical abnormalities that make a perineal biopsy of the prostate impossible according to routine clinical practice.

The target number of cases was set at a maximum of 20 patients, with recruitment being terminated once theoretical data saturation was reached. Patients were recruited at the Department of Urology- Medical Centre, Freiburg, Germany from May to August 2024 by one project-associated urologist (JH). The time point of recruitment was after the consultation to explain the surgical procedure. We followed a convenience sampling approach and selected patients via face-to-face consultation with the urologist. All included patients were then approached by phone, and subsequently, one researcher (UF) conducted semistructured telephone interviews before the biopsy was executed. All telephone interviews were audio-recorded with participants’ consent for subsequent transcription and analysis. The interview guide was developed via an iterative approach: the first version was drafted by UF and discussed within the research team. After some adjustments were made, the final version was approved by the team. The guide addressed the following topics: motivation to participate in the PRE-POET study, fears and emotions associated with the biopsy/cancer, coping strategies and measures against potential fears, thoughts about the ratio between diagnostic precision and side effects of the biopsy, tolerance toward side effects, motivation to participate in the planned RCT study, perceived aspects that might encourage participation, satisfaction with information about biopsy, patient-provider relationships and communication preferences (see supplementary material).

**2.2 Data analysis**

The audio material was transcribed verbatim by an external provider (amanu GmbH) and analyzed using the structuring content analysis approach [40]. To do this, two authors (UF and GJ) developed a coding scheme deductively from the interview guide. After pretesting the applicability of the coding scheme in one interview, it was intensively discussed by the two coders and refined consensually. During the coding procedure, the coders had the option to produce inductive (sub)codes where necessary. The data were coded in parallel and independently of each other by two authors (UF and GJ). Intercoder reliability (Kappa) was assessed and found to be low after the first coding round (Kappa=0.46). After consenting to indifferent coding patterns, we achieved a kappa of 0.73, which is considered good [41]. All coding steps were performed with MAXQDA 24. The standardized questionnaire data collected during the telephone interviewswere described by reporting frequencies via IBM SPSS Statistics 30.

This study was registered at the University Medical Center of Freiburg Clinical Trial Register (FRKS005027). The results are reported in compliance with the Consolidated Criteria for Reporting Qualitative Research (COREQ) checklist [42].

**2.3. Researcher characteristics**

UF is a male researcher working in the field of health services research. He holds a master’s degree in sociology and empirical social research and is experienced in conducting qualitative and quantitative research. GJ is a male researcher who also works in the field of health services research, holding a Master’s degree in psychology and is experienced in conducting qualitative and quantitative research. EF is a male professor in health services research and rehabilitation research with a focus on methodology. GD is a female physician. JH and AS, both of whom are male, are trained urologists with a clinical research focus on prostate cancer diagnostics. All the authors are native German. No personal relationships with the study subjects were established prior to study commencement.

1. **Results**
   1. **Sample characteristics**

In sum, we approached 18 patients (refusal rate: 33.3%). Non-participation was primarily due to a lack of interest or limited time availability for the telephone interview. We conducted 12 interviews with an average duration of 49 minutes (minimum = 31 minutes; maximum = 64 minutes). The interviewed men were 69.83 years old on average, and the majority (83.30%) lived in a steady partnership with an average household size of 2 persons (see Table 1). All the men were born in Germany, and one reported prior (nonprostate) cancer experience. With respect to the highest school degree, we covered no person without a degree but included individuals with lower, middle and higher school degrees, and the distribution seems to fit the observed age group [43]. The reported health status was good on average (Mean=2.92), as was the quality of life (Mean=4.33). The self-rated health literacy of the population was rather high, with a scale mean of 3.35 on average (see Table 1 for scale labels).

Table 1: Sample characteristics

| **Characteristic** | **Mean** | **SD** | **Min** | **Max** | **%** |
| --- | --- | --- | --- | --- | --- |
| **Age (years)** | 69.83 | 6.90 | 56 | 81 |  |
|  |  |  |  |  |  |
| **Partnership** |  |  |  |  |  |
| *yes* |  |  |  |  | 83.30 |
| *no* |  |  |  |  | 16.70 |
|  |  |  |  |  |  |
| **Household size (persons)** | 2.08 | 0.99 | 1 | 5 |  |
|  |  |  |  |  |  |
| **Highest school degree** |  |  |  |  |  |
| *Elementary/secondary school incl. polytechnic secondary school (8th, 9th grade)* |  |  |  |  | 41.70 |
| *Secondary school leaving certificate incl. polytechnic secondary school (10th grade)* |  |  |  |  | 25.00 |
| *High school diploma (Abitur), extended secondary school with 12th grade graduation* |  |  |  |  | 33.30 |
|  |  |  |  |  |  |
| **German born and citizenship** |  |  |  |  | 100 |
|  |  |  |  |  |  |
| **Prior cancer** |  |  |  |  |  |
| *yes* |  |  |  |  | 9.10 |
| *no* |  |  |  |  | 90.90 |
|  |  |  |  |  |  |
| **Health status** | 2.92 | 0.79 |  |  |  |
|  |  |  |  |  |  |
| **Quality of life** | 4.33 | 0.49 |  |  |  |
|  |  |  |  |  |  |
| **Self-rated health literacy** | 3.35 | 0.42 | 2.56 | 3.93 |  |
| *Health status was assessed on a 5-point scale: 1 "excellent", 2 "very good", 3 "good", 4 "average", 5 "bad". Quality of life was assessed on a 5-point scale: 1 "very bad", 2 "bad", 3 "neither/nor", 4 "good", 5 "very good". Health literacy was assessed using 16 items on a 4-point scale: 1 "very difficult", 2 "rather difficult", 3 "rather easy", 4 "very easy". An overall mean score was calculated.* | | | | | |

**Coding scheme**

The coding scheme covered six main categories and 17 subcategories (see Table 2).

Table 2: Coding scheme

| **Code** | ***Subcode*** |
| --- | --- |
| **Participation PRE-POET study** | *Reasons (pro vs. contra)* |
|  | *Motivation (egocentric vs. altruistic)* |
| **Participation future studies** |  |
|  | *Reasons (pro vs. contra)* |
|  | *Motivation (egocentric vs. altruistic)* |
|  | *Measures to incentivize study participation* |
| **Anxiety** |  |
|  | *Anxiety toward cancer* |
|  | *Anxiety toward death* |
| **Measures against anxiety** |  |
|  | *Patient resources (resilience, social support, internet use, etc.)* |
|  | *Provider resources (time, support services, etc.)* |
| **Biopsy: Side effect-diagnostic precision ratio** | |
|  | *Attitudes toward biopsy* |
|  | *Apparent information on biopsy side effects* |
|  | *Tolerance to side effects* |
|  | *Diagnostic precision* |
| **Patient-provider communication** |  |
|  | *Satisfaction & room for improvement* |
|  | *Relationship level & trust* |
|  | *Information sufficiency* |

**3.2. Motivation for study participation**

The patients expressed a strongly altruistic motivation to participate in the PRE-POET study. The interviewed men described that they might not directly profit from taking part in the study, but it might help other subsequent patients if they can contribute to producing new medical and scientific insights. Furthermore, they saw no personal disadvantages for them.

*“I am retired; I have time, and somehow, medicine has to move forward.” (P 12)*

*“If it helps me, if it helps others, then it is absolutely okay.” (P 11)*

*“I think that perhaps it might take the anxiety of any future patient […].” (P 8)*

The altruistic motivation also seems to be transferable to other future studies on prostate biopsy. After they explained how the planned study would be designed, they also observed no barriers in taking part in such a study, even though it might not have had a direct benefit for them. Motivation seemed to be associated with a high level of trust in the staff and its competences at the University Medical Center.

*“And I now assume that they already know in which area they have to prick in order to find it.” (P 6)*

*“So I do not see anything that speaks against it” (P 7)*

*“I say I'm always open to things like that. I have to say that very clearly. I always think the studies are good. I mean, if we did not have studies, we would not be where we are now in medicine; that has to be said quite clearly. There are always improvements.” (P 3)*

However, skepticism also arose in some cases due to the novelty of the approach. The doubt was related to greater insecurity that false-negative results might occur. In this case, personal harm was expected, which can be reduced only by valuable and transparent information and trust in the doctor.

*“Will you then perhaps have an MRI to check whether something has been overlooked? If this is checked afterwards, then I would take part. However, if you just take less and say: "Now let us see what happens", then probably not.” (P 12)*

*“Therefore, if you only do the [experimental] procedure […], then this should be combined with a follow-up check. Then, I would say immediately yes. However, if that is not the case, yes, then you always think: Ah, maybe they missed something. Or then you're somehow in a bit of an uncertain space.” (P 12)*

*“Therefore, how I feel about it is that I would prefer it if this study was completed and they already knew what was going on and then I could get through with the easier way.” (P 5)*

- 1. **Patient experience regarding involvement in future studies regarding biopsy decisions**

The interviewed patients characterized their roles as patient heterogeneously: While some expressed being lay people, others described their role in a more active and participatory manner. In many cases, patients decide to undergo biopsy on the basis of the advice of their attending physicians. Owing to fear, some patients explained that they tried to postpone the biopsy as long as possible.

*“I am now a complete medical layman” (P 11)*

*“And then you should actually become active, and that is what he [the urologist] recommended to me and I can understand that.” (P 9)*

*“However, also on the advice of the local urologist, who then said: You are welcome to get a second opinion at some point - that is why I went to the university clinic in Freiburg.” (P 7)*

*“I could decide for myself to watch, which is what I have always done now all the time. I always waited... watched. In addition, […] then I said: Yes, I have to […] do something” (P 1)*

- 1. **Patient fears and anxiety toward biopsy and cancer**

Patients were asked about their anxiety of the biopsy itself, toward possible side effects and toward consequences, i.e., the diagnosis of cancer. Fears toward the biopsy were apparent but not for all interviewed men. Some patients declared that their sorrows would be postponed to a moment, when more information was available. In terms of fears, patients reported the intertwining of physical consequences of the biopsy, e.g., pain, social consequences, e.g., effects on their sexual life, and psychological consequences, e.g., strain or fear of death.

*“Therefore, I think that is bearable for once. I'm not afraid of that now.” (P 11)*

*“Yes, in the past, before I even suspected that something was coming, I was more afraid that there would be a massive intervention. Massive for the man, for his sex life and so on. And then secondarily the fear that this carcinoma will take your life.” (P 10)*

*“Yes, that the cancer might then disappear through the cell through the body and metastasize, because hopefully I do not have any yet. That is the reason why I have always postponed the biopsy.” (P 1)*

*“Therefore, there's already the threat—I will call it a threat now—behind it, the perspective threat: this is now your death sentence.” (P 4)*

- 1. **Patient-related and clinical resources**

Although some fears were apparent related to the biopsy and its consequences, many patients also reported on the measures and resources they used to reduce strain. Strikingly, some patients emphasized the status of the attending physicians, their supposed expertise and the prestige of the University Hospital. Together with trust, the prestige of the University Hospital and its staff cause beliefs of being in good hands. However, patients also mention long waiting times to obtain reliable results. This phase of waiting is seen as an unnecessary stress factor.

*“This second biopsy was recommended by your professor at the time, in principle.” (P 7)*

*“The university hospital itself,* i.e.*, urology and so on, actually has a very good reputation.” (P 10)*

*“I do believe that the level of expertise is reasonably high, both among yourselves and among general practitioners. And that you simply say what's going on, what can be done.” (P 2)*

*“No, I'm not afraid at all because I'm at the university hospital.” (P 8)*

*“And then I ask myself, do not you have to be examined even faster or have this biopsy done, that if it were truly highly aggressive, this cell growth, […] that you do not wait too long.” (P 11)*

To overcome fears associated with biopsy, patients sought advice in their social environment and report on the distraction they find through conversations with others or through everyday activities. Some of them also reported about professionals in their social network whom they consulted. However, one man described that he does not want to involve his family unless there is no clear diagnosis communicated. Others reported trying not to think about the topic. Furthermore, information-seeking behavior was described not only as helpful but also as a potential source of greater concern.

*“And then I'm back in business this morning too. Then, I forget about myself because I have so much to do, which I also enjoy; otherwise, I would not do it. And then I forget about myself. It is only when I truly get to grips with it and all that, that I get scared at the back of my mind.” (P 1)*

*“Let us put it this way, the family definitely helps me with that” (P 8)*

*“And I'm somehow good at blocking it out. Sometimes I do not even think about it for days.” (P 12)*

*“Knowing more is not a bad thing, on the contrary. Sometimes like this, sometimes like this. I have already learned a lot of things, I have to say, if you know exactly what something is about.” (P 1)*

- 1. **Knowledge about and tolerance of side effects**

Knowledge about potential side effects differed. Some patients knew what they had to expect, and others reported little knowledge. The interviewed men described fears of pain, fears of consequences for their sexual life, expected incontinence and what it would mean for their personal life. However, they expressed that they would take many of the side effects into account to achieve the most reliable cancer-free diagnosis. As long as the side effects and impairments associated with biopsy are temporary, many patients are tolerant.

*“And if you had a bit of pain when sitting down, that would be bearable now. However, it would be inconvenient at the moment.” (P 12)*

*“It is not pleasant. However, okay, what's the point?” (P 7)*

*“Well, I would not like to have urinary incontinence and erectile dysfunction. But if it is only temporary, it is not a problem. Yes, but it would be bad in the long term” (P 9)*

*“Let us say I have permanent erectile dysfunction after this biopsy, but the biopsy says it is a cancer that is treatable, this or that, and I live on, then I accept the other.” (P 10)*

*“Erectile dysfunction is probably not the nicest thing, yes. However, if you do not do anything and you die afterwards, you will not benefit either.” (P 6)*

- 1. **Communication preferences, information sufficiency and satisfaction**

One topic that emerged repeatedly was the transparency of information and the need for open, trusting communication between the treating physicians and patients. Patients reported different impressions in the various phases before the biopsy, be it in relation to GPs, outpatient urologists or the consultation to explain the surgical procedure at the university hospital. Some patients emphasize the empathy and sympathy of their treating physicians and praise how much time they took for them. On the other hand, it is viewed negatively if the impressions of rush, time pressure and short attention spans are created. However, this does not necessarily arise from the consultation itself but can also be caused by the perception of long queues and full waiting rooms. Overall, although patients were satisfied with the information they received during the consultation, some of them stated in the interview that they still had questions and would like to have another consultation with the medical staff.

*“And this time pressure or this hectic pace or short notice and the way the questions were answered, that was not quite so pleasant for me personally.” (P 10)*

*“I was there ten to eight and then I had to queue for ages and got a bit stressed because I thought he did not have much time.” (P 9)*

*“Then, the doctor came straight away for a consultation, which was nice. The doctor's assistant was nice. I truly cannot complain. I think I was also given good medical information, and my questions were answered.” (P 8)*

*"Someone with whom I would have the feeling that they would be there more for my knowledge and psychological care than for medical care, and who would of course answer my questions and do something, but who would not want to train me as a specialist by telling me everything." (P 5)*

- 1. **Patient-relevant study attributes**

Patients reported dissatisfaction with long waiting times and the associated longer periods of uncertainty. Although delays were also caused by the fact that the biopsy was not scheduled by the patient, the interviewed men described that they found the waiting time after the decision to have a biopsy stressful and would like this phase to be shortened.

*“If it were acute now, then of course there would be a waiting time of, I guess, I do not know, but it is almost half a year until […] the biopsy is actually done. Therefore, it must have taken four months. In addition, if you truly have something existential now, then I think that is a long time.” (P 12)*

*“However, you cannot book anything, you cannot do anything, because you do not know: What happens next?” (P 12)*

*“And as I said, when you read on the internet that five weeks might be highly aggressive, then... well, I swallowed. Is five weeks perhaps too long to wait? Yes.” (P 11)*

When asked which factors are important for patients when planning a multicenter study, the topic of transparent communication was mentioned. Patients described the need for the advantages of the new procedure to be communicated clearly. From the patients' point of view, good communication creates a basis of trust that not only counteracts uncertainty and reduces fears but also increases the willingness to participate in studies to test new procedures.

*"Therefore, try to explain to the patient what is positive about it, how beneficial the new procedure is compared to the old one, how much gentler it is on the prostate than the old one." (P 10)*

*"The key word is trust, yes. And you can only gain that trust with a great consultation and with the right staff next to you." (P 10)*

*"And if it is all comprehensible, then why not?" (P 9)*

*"Well, I think that communication is probably the most important thing up front." (P 6)*

Ultimately, the patients described that their motive was to suffer as little harm as possible, e.g., in the form of side effects, and at the same time to receive the most reliable diagnoses possible.

*"... but for me as a patient, of course it is crucial that I know that they can investigate something sensibly and that they can then tell me afterwards that we can do something, that something can be done or not. So that is the decisive factor, and for me in my own assessment, whether I do it or not." (P 5)*

*"It would just be that the final result is just as reliable as the large biopsy, as you say. If you can guarantee me that, then yes." (P 2)*

*"You always want both. You want it to be safe and as little intervention as possible." (P 12)*

1. **Discussion**

This study explored themes relevant to patients undergoing prostate biopsy. The interview results indicate that many men experience fear related to the biopsy procedure. These fears were associated with potential side effects, including physical impairments and their consequences for social life. Additionally, the procedure's potential to detect cancer has raised concerns, leading to fears of having cancer and its associated mortality. This interconnectedness of physical, emotional and social impairments underlines the scope of the biopsychosocial model [44]. The role of perceived risk in relation to PSA testing and subsequent biopsy was described earlier in the literature [45]. A qualitative study from UK concluded that misunderstandings and inaccurate risk perception can prevent men from participating in PSA testing. In this study men took part in PSA testing without major fears due to a lack of symptoms. In contrast, men who did not undergo PSA testinganticipated that a PSA test is unnecessary due to the absence of urinary symptoms. This finding supports the hypothesis that fear and perception of symptoms are main drivers for participation in PSA testing. As consequence, the authors demand improved information about the lack of relationship between the risk of prostate cancer and urinary symptoms to support informed decision-making [45]. Based on our findings we come to the same conclusion since the necessity of transparent communication was emphasized by our patients multiple times.

Another UK study interviewing men after prostate biopsy emphasizes the importance of detailed information provision before the biopsy and the provision of opportunities to express fears [46]. This finding can also be supported by our results since some men expressed that the interviews alone and the associated opportunity to give their fears space provided them with support in the phase before the biopsy.

However, not all patients reported fear. Some men described the coping strategies they employ to remain calm. These coping mechanisms align with findings from other studies, such as information-seeking behavior, where patients use external resources to understand and process health information [47]. Social networks also play a role in reducing stress during emotionally challenging phases associated with the illness [48]. In addition to providing emotional support, these networks helped patients contextualize the health information they received. A study comparing primary care-based prostate cancer follow-up with specialist care revealed that patients identified several benefits in primary care follow-up: easier accessibility, proximity to home, and a more personal relationship with their respective healthcare providers [49].

The patients in our study were motivated by altruism to participate and expressed interest in future studies, such as the planned multicenter study on the diagnostic precision of prostate biopsy. From a theoretical point of view, this trait can be classified as research altruism, which we expect to be generally apparent in our sample and might lead to greater motivation to take part in future studies [50]. At this point, it should be noted that no financial incentives were used in this study, so this could be ruled out as a possible motivator. Research altruism can be a crucial prerequisite for successful implementation of PPI since it reflects – at least to some part - the personal motivation for patient participation in research.

However, the interviewed men also provided several insights that need to be considered when designing a study of high value for patients:

1. **Clear, Transparent, and Trustworthy Communication**: Patients view communication as a key feature that offers multiple benefits: The importance of good patient–provider communication in improving care quality, health outcomes, post-surgery support, impact on life, healthcare information and satisfaction has been highlighted in the literature [51–54]. This is supported by our patients, who felt that transparent communication would not only foster trust but also improve the understanding of the necessity of clinical studies.
2. **Perception of University and Specialized Clinical Centers**: On the one hand, the prestige and belief of highly educated professionals increased trust and gave patients a sense of being in good hands. On the other hand, long waiting times, long queues, and crowded waiting rooms cause stress and suggest that healthcare providers lack the resources to dedicate sufficient time to patients. Long waiting times are a common challenge in public health [55], but our patients reported that a reduction in waiting time could be a strong incentive to take part in a clinical study.
3. **Diagnostic Precision and Minimal Harm:** Patients discussed the importance of high diagnostic precision and minimizing harm to the body. It was difficult for the participants to articulate which side effects they would be willing to accept and at what cost. Generally, temporary side effects were deemed tolerable as long as diagnostic accuracy was ensured. These findings suggest that certainty in diagnosis is the primary priority for patients. However, it should be noted that these opinions were expressed before any potential consequences of the biopsy were known, meaning that participants had to anticipate their tolerance for side effects in advance. This anticipation makes it difficult to determine how patients experience side effects when they appear.

**4.1. Strengths and limitations**

One of the strengths of this study is the timing of the interviews, which were conducted between the doctor's information session and the actual biopsy. This ensured that the topic of the biopsy and its context were still fresh in the patients' minds; however, potential side effects had to be anticipated and could not yet be experienced and reported by patients. The exploratory qualitative approach allowed us to identify several patient-relevant factors in the context of prostate biopsy, including communication, trust, the prestige of the clinic, waiting times, diagnostic precision, and concerns about side effects and their consequences.

However, some limitations must be noted. The sample primarily represented older men, which is reflective of the general biopsy population; therefore, younger patients were not included in this analysis. Furthermore, we cannot include the perspectives of migrants, who are considered a vulnerable group with specific needs. D

1. **Conclusions**

The study contributes valuable knowledge about patients’ experiences, anxieties, and preferences preceding prostate biopsy. The findings highlight the multidimensional nature of the patient experience, encompassing physical, emotional, and social factors, and emphasize the need for a patient-centered approach in clinical decision-making and study design. The key themes for improving patient satisfaction and alleviating anxiety include transparent communication, trust in healthcare providers, the prestige of the clinic, waiting times, and minimizing the invasiveness of procedures while ensuring diagnostic precision.

The results underline the importance of incorporating the patient's perspective in future research, particularly in the development of new diagnostic approaches. Patients expressed a willingness to participate in clinical studies, driven primarily by altruism but also indicated that trust, transparent communication, and shorter waiting times could further enhance their participation.

**References**

1. Bray F, Laversanne M, Sung H, et al (2024) Global cancer statistics 2022: GLOBOCAN estimates of incidence and mortality worldwide for 36 cancers in 185 countries. CA: A Cancer Journal for Clinicians 74:229–263. https://doi.org/10.3322/caac.21834

2. Ferlay J, Ervik M, Lam F, Laversanne M, Colombet M, Mery L, Piñeros M, Znaor A, Soerjomataram I, Bray F Global Cancer Observatory: Cancer Today. Lyon, France: International Agency for Research on Cancer. https://gco.iarc.who.int/today. Accessed 25 Apr 2025

3. Leitlinienprogramm Onkologie (Deutsche Krebsgesellschaft, Deutsche Krebshilfe, AWMF): S3-Leitlinie Prostatakarzinom, Langversion 7.0, 2024, AWMF-Registernummer: 043-022OL https://www.leitlinienprogramm-onkologie.de/leitlinien/prostatakarzinom/; Accessed [18.12.2024]

4. P. Cornford, D. Tilki, E. Briers, G. De Meerleer, M. De Santis, D. Eberli, S. Gillessen Sommer, A. Henry,, J. Oldenburg, D. Oprea-Lager, M. Roberts, O, Rouvière, I.G. Schoots, J. Stranne, R.C.N. Van Den Bergh, G. Van Leenders, I.M. Van Oort, T. Wiegel, Guidelines Associates: P.K-F.ChiuC, et al. EAU Guidelines. Edn. presented at the EAU Annual Congress Milan 2023. ISBN 978-94-92671-19-6. EAU Guidelines Office, Arnhem, the Netherlands.

5. Ahmed HU, El-Shater Bosaily A, Brown LC, et al (2017) Diagnostic accuracy of multi-parametric MRI and TRUS biopsy in prostate cancer (PROMIS): a paired validating confirmatory study. The Lancet 389:815–822. https://doi.org/10.1016/S0140-6736(16)32401-1

6. Fütterer JJ, Briganti A, De Visschere P, et al (2015) Can Clinically Significant Prostate Cancer Be Detected with Multiparametric Magnetic Resonance Imaging? A Systematic Review of the Literature. Eur Urol 68:1045–1053. https://doi.org/10.1016/j.eururo.2015.01.013

7. Muthigi A, George AK, Sidana A, et al (2017) Missing the Mark: Prostate Cancer Upgrading by Systematic Biopsy over Magnetic Resonance Imaging/Transrectal Ultrasound Fusion Biopsy. The Journal of Urology. https://doi.org/10.1016/j.juro.2016.08.097

8. Woo S, Suh CH, Kim SY, et al (2017) Diagnostic Performance of Prostate Imaging Reporting and Data System Version 2 for Detection of Prostate Cancer: A Systematic Review and Diagnostic Meta-analysis. European Urology 72:177–188. https://doi.org/10.1016/j.eururo.2017.01.042

9. Sigle A, Jilg CA, Kuru TH, et al (2021) Evaluation of the Ginsburg Scheme: Where Is Significant Prostate Cancer Missed? Cancers (Basel) 13:2502. https://doi.org/10.3390/cancers13102502

10. Tosoian JJ, Mamawala M, Epstein JI, et al (2020) Active Surveillance of Grade Group 1 Prostate Cancer: Long-term Outcomes from a Large Prospective Cohort. European Urology 77:675–682. https://doi.org/10.1016/j.eururo.2019.12.017

11. Bryk DJ, Llukani E, Taneja SS, et al (2017) The Role of Ipsilateral and Contralateral Transrectal Ultrasound-guided Systematic Prostate Biopsy in Men With Unilateral Magnetic Resonance Imaging Lesion Undergoing Magnetic Resonance Imaging-ultrasound Fusion-targeted Prostate Biopsy. Urology 102:178–182. https://doi.org/10.1016/j.urology.2016.11.017

12. Hansen NL, Barrett T, Lloyd T, et al (2020) Optimising the number of cores for magnetic resonance imaging-guided targeted and systematic transperineal prostate biopsy. BJU International 125:260–269. https://doi.org/10.1111/bju.14865

13. Wu Q, Tu X, Jiang J, et al (2024) Is ipsilateral systematic biopsy combined with targeted biopsy the optimal substitute for bilateral systematic biopsy combined with targeted biopsy: A systematic review and meta-analysis. Urologic Oncology: Seminars and Original Investigations. https://doi.org/10.1016/j.urolonc.2024.11.023

14. Brisbane WG, Priester AM, Ballon J, et al (2022) Targeted Prostate Biopsy: Umbra, Penumbra, and Value of Perilesional Sampling. European Urology 82:303–310. https://doi.org/10.1016/j.eururo.2022.01.008

15. Desai M (2020) Recruitment and retention of participants in clinical studies: Critical issues and challenges. Perspect Clin Res 11:51. https://doi.org/10.4103/picr.PICR_6_20

16. Bouzalmate-Hajjaj A, Cano-Ibáñez N, Khan KS, et al (2023) Patient and public involvement in lifestyle randomized controlled trials: a systematic review. J Public Health (Berl). https://doi.org/10.1007/s10389-023-02148-0

17. Simons G, Jones H, Clarke I, et al (2024) Patient and public involvement in the design of an international clinical trial: real world experience. Res Involv Engagem 10:117. https://doi.org/10.1186/s40900-024-00642-7

18. Greenhalgh T, Hinton L, Finlay T, et al (2019) Frameworks for supporting patient and public involvement in research: Systematic review and co‐design pilot. Health Expectations 22:785–801. https://doi.org/10.1111/hex.12888

19. Staniszewska S, Haywood KL, Brett J, Tutton L (2012) Patient and Public Involvement in Patient-Reported Outcome Measures: Evolution Not Revolution. The Patient: Patient-Centered Outcomes Research 5:79–87. https://doi.org/10.2165/11597150-000000000-00000

20. Groene O (2012) Patient and Public Involvement in Developing Patient-Reported Outcome Measures: Indispensable, Desirable, Challenging. The Patient: Patient-Centered Outcomes Research 5:75–77. https://doi.org/10.2165/11597370-000000000-00000

21. Beyer K, MacLennan SJ, Moris L, et al (2021) The Key Role of Patient Involvement in the Development of Core Outcome Sets in Prostate Cancer. European Urology Focus 7:943–946. https://doi.org/10.1016/j.euf.2021.09.008

22. Berry B, Parry MG, Sujenthiran A, et al (2020) Comparison of complications after transrectal and transperineal prostate biopsy: a national population‐based study. BJU International 126:97–103. https://doi.org/10.1111/bju.15039

23. Merriel SWD, Hardy V, Thompson MJ, et al (2020) Patient-Centered Outcomes From Multiparametric MRI and MRI-Guided Biopsy for Prostate Cancer: A Systematic Review. Journal of the American College of Radiology 17:486–495. https://doi.org/10.1016/j.jacr.2019.08.031

24. Fainberg J, Gaffney CD, Pierce H, et al (2021) Erectile Dysfunction is a Transient Complication of Prostate Biopsy: A Systematic Review and Meta-Analysis. The Journal of Urology. https://doi.org/10.1097/JU.0000000000001398

25. Zattoni F, Rajwa P, Miszczyk M, et al (2024) Transperineal Versus Transrectal Magnetic Resonance Imaging–targeted Prostate Biopsy: A Systematic Review and Meta-analysis of Prospective Studies. European Urology Oncology 7:1303–1312. https://doi.org/10.1016/j.euo.2024.07.009

26. Miah S, Eldred-Evans D, Simmons LAM, et al (2018) Patient Reported Outcome Measures for Transperineal Template Prostate Mapping Biopsies in the PICTURE Study. J Urol 200:1235–1240. https://doi.org/10.1016/j.juro.2018.06.033

27. Gulati R (2022) Reducing Prostate Cancer Overdiagnosis. N Engl J Med 387:2187–2188. https://doi.org/10.1056/NEJMe2214658

28. Gaffney C, Vertosick EE, Carlsson SV, et al (2023) A brief mind-body intervention to reduce pain and anxiety during prostate needle biopsy: A randomized, controlled trial with two-staged consent. JCO 41:e17092–e17092. https://doi.org/10.1200/JCO.2023.41.16_suppl.e17092

29. Stanley E, Cradock A, Bisset J, et al (2016) Impact of sensory design interventions on image quality, patient anxiety and overall patient experience at MRI. BJR 89:20160389. https://doi.org/10.1259/bjr.20160389

30. Kasivisvanathan V, Rannikko AS, Borghi M, et al (2018) MRI-Targeted or Standard Biopsy for Prostate-Cancer Diagnosis. N Engl J Med 378:1767–1777. https://doi.org/10.1056/NEJMoa1801993

31. De Sio M, D’armiento M, Di Lorenzo G, et al (2005) The need to reduce patient discomfort during transrectal ultrasonography‐guided prostate biopsy: what do we know? BJU International 96:977–983. https://doi.org/10.1111/j.1464-410X.2005.05736.x

32. Chesnut GT, Zareba P, Sjoberg DD, et al (2020) Patient-reported pain, discomfort, and anxiety during magnetic resonance imaging-targeted prostate biopsy. Can Urol Assoc J 14:E202–E208. https://doi.org/10.5489/cuaj.6102

33. Protopapa E, Van Der Meulen J, Moore CM, Smith SC (2017) Patient‐reported outcome ( PRO ) questionnaires for men who have radical surgery for prostate cancer: a conceptual review of existing instruments. BJU International 120:468–481. https://doi.org/10.1111/bju.13896

34. Van Hemelrijck M, Sparano F, Moris L, et al (2020) Harnessing the patient voice in prostate cancer research: Systematic review on the use of patient‐reported outcomes in randomized controlled trials to support clinical decision‐making. Cancer Medicine 9:4039–4058. https://doi.org/10.1002/cam4.3018

35. Fowler FJ, Barry MJ, Walker-Corkery B, et al (2006) The impact of a suspicious prostate biopsy on patients’ psychological, socio-behavioral, and medical care outcomes. J Gen Intern Med 21:715–721. https://doi.org/10.1111/j.1525-1497.2006.00464.x

36. Mao J, Dai Y, Wang L, et al (2022) “Is it painful”? A qualitative study on experiences of patients before prostate needle biopsy. BMJ Open 12:e056619. https://doi.org/10.1136/bmjopen-2021-056619

37. Smith TW, Schapiro B (2021) The International Social Survey Program Modules on Religion, 1991–2018. International Journal of Sociology 51:337–344. https://doi.org/10.1080/00207659.2021.1976471

38. World Health Organization (2025) WHOQOL-BREF. https://www.who.int/tools/whoqol/whoqol-bref/docs/default-source/publishing-policies/whoqol-100-guidelines/translation-methodology. Accessed 14 Oct 2025

39. Jordan S, Hoebel J (2015) Gesundheitskompetenz von Erwachsenen in Deutschland: Ergebnisse der Studie „Gesundheit in Deutschland aktuell“ (GEDA). Bundesgesundheitsbl 58:942–950. https://doi.org/10.1007/s00103-015-2200-z

40. Mayring P (2008) Qualitative Inhaltsanalyse - Grundlagen und Techniken, 10th ed. Beltz, Weinheim

41. Galle M (2021) Unterrichtszentrierte Schulentwicklung: Schulen auf dem Weg zu einer personalisierten Gestaltung von Lehr- und Lernprozessen. Springer Fachmedien Wiesbaden, Wiesbaden

42. Tong A, Sainsbury P, Craig J (2007) Consolidated criteria for reporting qualitative research (COREQ): a 32-item checklist for interviews and focus groups. International Journal for Quality in Health Care 19:349–357. https://doi.org/10.1093/intqhc/mzm042

43. Autorengruppe Bildungsberichterstattung der Bundeszentrale für politische Bildung (2012) Bildung in Deutschland 2012. https://www.bpb.de/themen/bildung/dossier-bildung/159282/welche-abschluesse-erreichten-schueler-frueher-und-heute/. Accessed 23 Jan 2025

44. Engel GL (1977) The Need for a New Medical Model: A Challenge for Biomedicine. Science 196:129–136. https://doi.org/10.1126/science.847460

45. Avery KNL, Blazeby JM, Lane JA, et al (2008) Decision-Making about PSA Testing and Prostate Biopsies: A Qualitative Study Embedded in a Primary Care Randomised Trial. European Urology 53:1186–1193. https://doi.org/10.1016/j.eururo.2007.07.040

46. Chapple AB, Ziebland S, Brewster S, Mcpherson A (2007) Patients? perceptions of transrectal prostate biopsy: a qualitative study. Eur J Cancer Care 16:215–221. https://doi.org/10.1111/j.1365-2354.2006.00766.x

47. Steptoe A, Sutcliffe I, Allen B, Coombes C (1991) Satisfaction with communication, medical knowledge, and coping style in patients with metastatic cancer. Social Science & Medicine 32:627–632. https://doi.org/10.1016/0277-9536(91)90141-X

48. Kamarck TW, Annunziato B, Amateau LM (1995) Affiliation Moderates the Effects of Social Threat on Stress-Related Cardiovascular Responses: Boundary Conditions for a Laboratory Model of Social Support. Psychosomatic Medicine 57:183–194. https://doi.org/10.1097/00006842-199503000-00011

49. Wollersheim BM, Van Asselt KM, Akdemir E, et al (2021) Core elements for the implementation of primary care-based prostate cancer follow-up: A process evaluation of a randomized controlled trial. JCO 39:242–242. https://doi.org/10.1200/JCO.2020.39.28_suppl.242

50. Carrera JS, Brown P, Brody JG, Morello-Frosch R (2018) Research altruism as motivation for participation in community-centered environmental health research. Social Science & Medicine 196:175–181. https://doi.org/10.1016/j.socscimed.2017.11.028

51. Jahan F, Siddiqui H (2019) Good Communication between Doctor-Patient Improves Health Outcome. EJMED 1:. https://doi.org/10.24018/ejmed.2019.1.4.84

52. Breyer M, Shockley L (2019) Patient Communication. In: Moreira ME, French AJ (eds) Communication in Emergency Medicine. Oxford University Press, pp 45–62

53. Teutsch C (2003) Patient–doctor communication. Medical Clinics of North America 87:1115–1145. https://doi.org/10.1016/S0025-7125(03)00066-X

54. Darwish M, Nandy S, Willis S, et al (2024) Surgical patient-reported experience measures and qualitative experience studies: systematic review. BJS Open 9:zrae142. https://doi.org/10.1093/bjsopen/zrae142

55. McIntyre D, Chow CK (2020) Waiting Time as an Indicator for Health Services Under Strain: A Narrative Review. INQUIRY 57:0046958020910305. https://doi.org/10.1177/0046958020910305
